# Extracellular *Onchocerca*-derived small RNAs in host nodules and blood

**DOI:** 10.1186/s13071-015-0656-1

**Published:** 2015-01-27

**Authors:** Juan F Quintana, Benjamin L Makepeace, Simon A Babayan, Alasdair Ivens, Kenneth M Pfarr, Mark Blaxter, Alexander Debrah, Samuel Wanji, Henrietta F Ngangyung, Germanus S Bah, Vincent N Tanya, David W Taylor, Achim Hoerauf, Amy H Buck

**Affiliations:** Centre for Immunity, Infection and Evolution, Ashworth Laboratories, University of Edinburgh, West Mains Road, Edinburgh, UK; Institute of Infection and Global Health, University of Liverpool, Liverpool, Merseyside UK; Institute of Biodiversity, Animal Health and Comparative Medicine, College of Medical, Veterinary and Life Sciences, University of Glasgow, Glasgow, UK; Institute of Medical Microbiology, Immunology and Parasitology, University Hospital of Bonn, Bonn, Germany; Kumasi Centre for Collaborative Research (KCCR) and Kwame Nkrumah University of Science and Technology, Kumasi, Ghana; Research Foundation in Tropical Diseases and Environment and University of Buea, Buea, Cameroon; Institut de Recherche Agricole pour le Développement, Regional Centre of Wakwa, Ngaoundéré, Cameroon; Cameroon Academy of Sciences, Yaoundé, Cameroon; Division of Pathway Medicine, School for Biomedical Sciences, University of Edinburgh, Little France, Edinburgh, UK

**Keywords:** microRNAs, Extracellular RNA, Filarial nematode, Onchocerciasis, Host-pathogen

## Abstract

**Background:**

microRNAs (miRNAs), a class of short, non-coding RNA can be found in a highly stable, cell-free form in mammalian body fluids. Specific miRNAs are secreted by parasitic nematodes in exosomes and have been detected in the serum of murine and dog hosts infected with the filarial nematodes *Litomosoides sigmodontis* and *Dirofilaria immitis*, respectively. Here we identify extracellular, parasite-derived small RNAs associated with *Onchocerca* species infecting cattle and humans.

**Methods:**

Small RNA libraries were prepared from total RNA extracted from the nodule fluid of cattle infected with *Onchocerca ochengi* as well as serum and plasma from humans infected with *Onchocerca volvulus* in Cameroon and Ghana. Parasite-derived miRNAs were identified based on the criteria that sequences unambiguously map to hairpin structures in *Onchocerca* genomes, do not align to the human genome and are not present in European control serum.

**Results:**

A total of 62 mature miRNAs from 52 distinct pre-miRNA candidates were identified in nodule fluid from cattle infected with *O. ochengi* of which 59 are identical in the genome of the human parasite *O. volvulus*. Six of the extracellular miRNAs were also identified in sequencing analyses of serum and plasma from humans infected with *O. volvulus*. Based on sequencing analysis the abundance levels of the parasite miRNAs in serum or plasma range from 5 to 127 reads/per million total host miRNA reads identified, comparable to our previous analyses of *Schistosoma mansoni* and *L. sigmodontis* miRNAs in serum. All six of the *O. volvulus* miRNAs identified have orthologs in other filarial nematodes and four were identified in the serum of mice infected with *L. sigmodontis*.

**Conclusions:**

We have identified parasite-derived miRNAs associated with onchocerciasis in cattle and humans. Our results confirm the conserved nature of RNA secretion by diverse nematodes. Additional species-specific small RNAs from *O. volvulus* may be present in serum based on the novel miRNA sequences identified in the nodule fluid. In our analyses comparison to European control serum illuminates the scope for false-positives, warranting caution in criteria that should be applied to identification of biomarkers of infection.

**Electronic supplementary material:**

The online version of this article (doi:10.1186/s13071-015-0656-1) contains supplementary material, which is available to authorized users.

## Background

Small non-coding RNAs (sncRNAs) have emerged as important regulators of many processes in animals, from development to immunity. MicroRNAs (miRNAs) are the best characterized class of sncRNA which operate by guiding the RNA-induced silencing complex (RISC) to specific messenger RNAs (mRNAs) inside cells, where they inhibit translation and de-stabilize the targeted mRNAs [1]. In parasitic nematodes and flatworms, miRNAs have been shown to have core roles in the physiology of development, differentiation and homeostasis and potentially drug resistance [2]. Studies in the last 7 years have demonstrated that miRNAs can also exist in a cell-free form in extracellular fluids, where they may play endocrine signalling roles, reviewed in [3]. For parasitic species, interacting with this signalling system offers another potential mechanism of host manipulation. We and others have identified miRNAs from nematodes and trematodes in the serum of infected animals [4-6] and initial studies with *S. mansoni* demonstrated the utility of these molecules in distinguishing uninfected and infected humans [4]. The exact origin of these circulating parasite RNAs is unknown, but proteomic analysis of *Dicrocoelium dendriticum* suggests RNAs are associated with exosomes secreted from the parasite surface [7] and it is possible that previously described microvesicles in schistosomes could also contain RNA [8]. Recently we showed that miRNAs are packaged within vesicles secreted by the gastrointestinal nematode *Heligmosomoides polygyrus* and that these derive from the intestine of the nematode. These secreted vesicles (and their cargoes) suppress Th2 innate immune responses *in vivo* and the miRNAs within them are transferred to host cells *in vitro* [9]. Homologues of some of the miRNAs secreted by *H. polygyrus* miRNAs were also found in serum of hosts infected with the filarial nematodes *Litomosoides sigmodontis* [9] and *Dirofilaria immitis* [5]. The miRNAs secreted by nematodes and platyhelminth parasites may be a new axis of host-parasite interaction. Here we characterize the extracellular, parasite-derived miRNAs associated with the important human disease onchocerciasis*.*

Filarial infections currently affect over 150 million people in tropical and subtropical regions [10], with *Onchocerca volvulus* accounting for approximately 30.4 million [11] of which more than 99% occur in Africa. Onchocerciasis is characterised by skin disease, which can be very severe, and is also the second leading cause of infectious blindness. *Onchocerca ochengi*, a filarial parasite of cattle, is the closest relative of *O. volvulus,* with which it is sympatric*,* and shares several key features with the human parasite [12,13]. Specifically, *O. ochengi* induces the formation of onchocercomata with very similar histological structure to human nodules [14], and both *O. ochengi* and *O. volvulus* present comparable mating behaviour within the nodules and subsequent Mf production, leading to a patent infection over a similar timescale [12]. The phylogenetic closeness means that the two species have very similar genomes, and thus very closely related (sometimes identical) antigens are present in both. There is evidence of cross-protection [15]. Therefore, *O. ochengi* represents the most relevant experimental model to understand the crosstalk between the parasite and the host in the context of onchocerciasis.

Since 1989, ivermectin has been used in mass drug administration (MDA) programmes to control onchocerciasis in Africa and Latin America. Following the success of the Onchocerciasis Elimination Program for the Americas, which has used MDA of ivermectin alone to abrogate transmission in most endemic foci, the goal of the African Programme for Onchocerciasis Control (APOC; which covers a vastly greater area) has shifted from control to eradication [13]. However, major challenges to this endeavour remain, such as the emergence of ivermectin resistance [16], the potential for severe adverse reactions to ivermectin in loiasis-endemic areas [17], and significant limitations in the accurate and rapid diagnosis of infection [18]. Currently, diagnosis relies on identification of microfilariae in skin snips, which are laborious and notoriously insensitive; additionally, this procedure can cause considerable discomfort. The availability of immunoassays such as the Ov16 serological test [19] has greatly enhanced the ability to detect residual transmission or the re-emergence of infection by using young children as “sentinels”; however, the longevity of immune responses in onchocerciasis renders this assay unsuitable as a tool to confirm elimination of infection from adults [20].

Detection of parasite DNA in a wide variety of bodily fluids by either polymerase chain reaction (PCR) or high-throughput deep sequencing has proven to be successful in the diagnosis of infections caused by *S. mansoni*, gastrointestinal parasitic nematodes [21] and *Leishmania* [22], among others. DNA-based tests thus represent an alternative diagnostic platform to conventional parasitological or antigen-based assays. sncRNAs are another class of diagnostic biomarker that can be amplified and are detectable by qRT-PCR. miRNAs are generally ~22 nt in length and have been detected outside of cells in many mammalian body fluids indicating that these molecules can be rendered highly stable and protected against extreme conditions (i.e. low pH, degradation by extracellular RNases, etc.) [23]. The functional significance of their extracellular existence is still elusive [3,23] but they have been shown to act locally in cell-to-cell communication in mammalian systems [3] and can also be moved from parasite to host via exosomes [9].

Here we report the detection and identification of *Onchocerca* spp. miRNAs from bovine nodular fluid *ex vivo* and the detection of a subset of these molecules in the serum and plasma of human onchocerciasis patients from Ghana and Cameroon. Several of these miRNAs are orthologs of (and in some cases have identical sequence to) those previously identified in serum of mice infected with *L. sigmodontis* as well as miRNAs secreted by *H. polygyrus in vitro*. Our findings indicate that miRNA secretion by nematodes is conserved in *Onchocerca* species.

## Methods

### *O. ochengi* nodule fluid

Bovine skins containing numerous *O. ochengi* onchocercomata were obtained from Ngaoundéré abattoir, Adamawa Region, Cameroon [24]. Freshly excised nodules were screened visually, and discarded if hard or discoloured, which are signs of calcification. Nodules were rinsed in PBS, dried thoroughly, and pricked with a 21G hypodermic needle. The nodules were gently squeezed, and the expressed fluid (~0.5 μl) collected with a micropipette, pooling from >10 nodules per biological replicate. The fluid was spun at 500 g for 5 min to pellet any cellular material or Mf, and the supernatant was stored at -80°C. The samples were shipped to the UK on dry ice and remained frozen prior to analysis.

### Human serum samples

Archived human plasma from *O. volvulus* infected and uninfected volunteers was collected as part of a European Union Seventh Framework Programme Research grant, contract 131242 “Enhanced Protective Immunity Against Filariasis (EPIAF)”, (http://cordis.europa.eu/project/rcn/94066_en.html). Infected individuals were those with palpable nodules and microfilaridermia by skin snip. Uninfected individuals were defined as persons with no palpable nodules and microfilariae negative skin snips. Serum or EDTA plasma was collected as previously described [25].

### Ethics statement

The Committee on Human Research Publication and Ethics at the University of Science and Technology in Kumasi, Ghana, and the Ethics Committee at the University of Bonn, Germany approved the use of archived plasma samples. Collection of sera from onchocerciasis patients in Cameroon was approved by the Cameroon Ethics Committee and the Ministry of Public Health as part of the EU FP7 contract 131242 (EPIAF) and in compliance with the Helsinki declaration on the use of humans in biomedical research. Prior to recruitment, the nature and objectives of the study were explained to potential participants and those who agreed to take part in the study signed a consent form while an assent was obtained from parents or guardians of children who were enrolled in the study. Participation was voluntary.

### RNA extraction and library preparation

RNA was extracted from 20 μL of pooled nodule fluids from cattle (*O. ochengi* infection), 200 μL of serum pooled from 12 infected individuals in Cameroon (pooled prior to RNA extraction), or pooled from equal volumes of RNA extracted from 13 infected or 13 uninfected individuals in Ghana (total equivalent of 50 μL plasma). Samples were shipped in liquid nitrogen and stored at −80°C for 3–4 years (Cameroon samples) or 5 years (Ghana samples). The serum was thawed on ice and serum or plasma was spun down at 16,000 g for 5 min at 4°C to remove any additional cell debris. The cleared serum was then transferred to a new 2 mL Eppendorf tube and RNA extracted using the miRCURY RNA isolation kit for Biofluids (Exiqon) according to manufacturers’ protocols. In both cases, RNA was eluted in 50 μL of 0.1 mM EDTA. RNA was stored at −20°C prior to further analysis. The relative small RNA content from these samples was determined with 1 μL of total RNA on a Bioanalyzer small RNA chip (Agilent).

Before proceeding with small RNA library preparation from serum or plasma RNA, samples were cleaned up as in [26]. Briefly, 50 μL of eluted RNA was diluted to 100 μL with Nuclease-free MiliQ water followed by addition of 1 μL glycoblue 15 mg/ml (Life technologies), 60 μL of Sodium acetate 3 M pH 5.2 (AppliChem) and 500 μL of ethanol 100%. The RNA was precipitated for 30 min at −80°C then spun at 16,000 g for 30 min at 4°C and washed twice with 75% Ethanol. The pellets were air-dried at room temperature for 15 minutes and re-suspended in 8 μL of 0.1 mM EDTA pH 8.0.

For the analysis of small RNA content in these nodule fluids or human serum and plasma, libraries were prepared from total RNA using the Illumina TruSeq small RNA Preparation kit, according to the manufacturers’ protocol, and using 1:10 dilution of adapters. PCR products of the expected molecular weight (140–160 bp) were size selected and sequenced on an IlluminaHiSeq2500 instrument using v3 reagents in Edinburgh Genomics (http://genomics.ed.ac.uk/).

### Bioinformatic analysis

All libraries were analysed by first clipping the 3′ sRNA adapter using cut adapt [27], searching for at least a 6 base match to the adapter sequence. For analysis of small RNAs, only sequences that contained the adapter, were >16 nt in length, and were present at ≥ 2 copies were retained for further analysis. Sequences were analyzed for alignment to bovine (ftp://ftp.ensembl.org/pub/release-71/fasta/bos_taurus/dna/Bos_taurus.UMD3.1.71.dna.toplevel.fa.gz), human (version hg19), *O.ochengi* (version 1.1; from http://onchocerca.nematod.es; unpublished genome sequence from M. Blaxter, B. Makepeace and colleagues) or *O. volvulus* (ftp://ftp.sanger.ac.uk/pub/project/pathogens/Onchocerca/volvulus/OVOC.V3.fa) using bowtie [28], requiring perfect matches along the full length of the sequence. Sequences were analyzed for known classes of RNA based on Rfam [29] (version 11, obtained from ftp://ftp.sanger.ac.uk/pub/databases/Rfam/11.0/). The best hit with at most two mismatches was used to classify the reads. Analysis of miRNA content was carried out using miRdeep2 [30] with the following settings: 1) reads map perfectly to the genome, 2) cut off -v 1, 3) employing the “-s option” using all mature sequences from mirbase (version 20) [31], 4) only read lengths (18 to 30 nt) were analyzed. Folding analyses of the novel pre-miRNAs detected in nodule fluids were carried out using the RNAfold Vienna package with default settings [32].

## Results

### *O. ochengi* small RNAs are present in bovine nodule fluid

**Figure 1:**
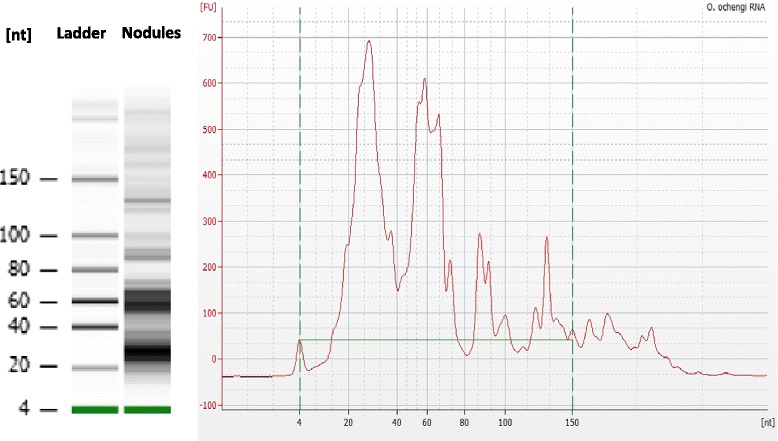
**Small RNA profile of nodules from cattle infected with** ***O. ochengi*** **.** Gel and electropherogram of total RNA (1 uL) from nodule fluid based on a small RNA chip (Bioanalyzer).

RNA from the fluid of nodules of cattle infected with *O. ochengi* was analysed for small RNA content using the Agilent Bioanalyzer. Two populations were observed between 20–30 nt and 50–70 nt (Figure 1), similar to the small RNA profile previously observed for the *in vitro* secretion products of the gastrointestinal nematode *H. polygyrus* [9]. Small RNA libraries were prepared and sequenced to identify the small RNAs between 17 and 40 nt and analysed as previously described [4]. Importantly, to avoid analysis of sequencing artefacts, reads that were present in < 2 copies were discarded and according to default criteria of miRdeep2 alignments only reads > 16 nt were analysed. To further increase confidence in our assignments, we required that reads contained the 3′ adapter and aligned along their full length to the bovine or *O. ochengi* draft genomes. Since some small RNA sequences can be post-transcriptionally edited this method may miss true positives. Following these criteria, a total of 15,043,191 reads were analysed of which 11,905,898 aligned to the bovine genome and 151,332 aligned to the *O. ochengi* genome (Table 1). A total of 6,301 reads that could be equivalently aligned to both genomes were not included in the analysis since their origin could not be determined.

**Table 1:** **Bovine and** ***O.ochengi*** **small RNA classification in nodules**

**Trimmed reads**	**15022278**
**Bovine genome match**	**11912199**
**Unambigious**	11905898
rRNA	20836
tRNA	11335037
Y RNA	51307
Other Rfam	25716
miRNA	382791
***O. ochengi*** **genome match**	**157633**
**Unambigious**	151332
rRNA	2616
tRNA	120733
Y RNA	0
Other Rfam	2344
miRNA	11455

**Figure 2:**
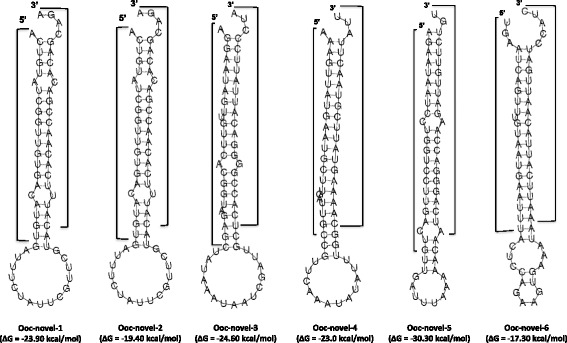
**Novel** ***O.ochengi*** **pre-miRNAs identified in bovine nodule fluid.** Predicted secondary structure of the novel pre-miRNAs identified by presence of reads mapping to both 5p and 3p arms (indicated in brackets). The minimum free energies (MFE) are indicated according to prediction by RNAfold.

The small RNA content was initially classified based on sequence identity (assessed using BLAST [33]) to known RNA classes in Rfam and revealed a predominance of tRNA fragments (Table 1), as previously observed in other extracellular fluids [4,34]. Identification of miRNAs was carried out using miRdeep2 which not only identifies matches to miRNAs already present in miRbase but also identifies novel miRNAs based on the ability of the reads to map to potential hairpins in the genome [30]. From this analysis a total of 62 mature miRNAs were identified including 23 that are identical to previously described nematode miRNAs (primarily *Ascaris suum* and *Brugia malayi*), 18 that are only identical in their seed sites (nucleotides 2 to 8) to other nematode miRNAs, and 21 of which did not share homology in their seed sites (Additional file 1: Table S1). From these analyses we identify 16 novel pre-miRNA candidates that are not homologs of other known nematode pre-miRNAs. Six of these have reads mapping specifically to both arms of the hairpin with 3′ overhangs (Figure 2) and we therefore assign confidence to their classification as Dicer-derived miRNAs. To determine whether these small RNAs are conserved in the closely related human parasite *O. volvulus*, miRdeep2 analysis of the reads sequenced in the *O. ochengi* nodule material was carried out using the *O. volvulus* draft genome as the mapping substrate, allowing for up to 2 mismatches. All sequences aligned perfectly and derived from hairpins, apart from three miRNAs (Ooc-novel-3-3p, Ooc-novel-15, Ooc-miR-49) where a 1 nt mismatch was present (Additional file 1: Table S1).

### Six parasite-derived miRNAs are detected in serum or plasma of humans infected with *O. volvulus*

To date very little is known about the factors that dictate the stability of extracellular RNA in fluids or whether and how these traffic within the body. The nodules are highly vascularized [35] and provide a direct route to the blood system for parasite-derived molecules. To determine whether *O. volvulus*-derived miRNAs are present in serum and plasma we carried out two parallel analyses of samples pooled from infected humans obtained from two geographically relevant regions in Africa, Cameroon [36] and Ghana [37], and compared these to pooled endemic or European controls. At present there is no standardized way to assess the integrity or quality of RNA extracted from serum since the intact large ribosomal RNAs (rRNAs) are not present. However we observed a distinct population of small RNA that is 20–30 nt in length based on Bioanalyzer analyses. This population was more prominent when using the Exiqon biofluids extraction kit compared to the Qiagen miRNA easy kit (Additional file 2: Figure S1). Small RNA sequencing libraries were prepared, sequenced and analysed as above. A total of 10–25 million reads were analysed per sample, of which 39-51% mapped to the human genome and 1.0-1.5% mapped to the draft *O. volvulus* genome. The majority of the reads from human serum that mapped to *O. volvulus* were identified as rRNA fragments, but these were detected at comparable levels in European control and infected samples. It is possible these are of human origin but are edited or that they derive from other organisms or dietary sources as shown in several studies [38,39]. Similarly the source of many reads that map to *O. volvulus* genome and are annotated as tRNA fragments [34] cannot be reliably assigned (Table 2).

**Table 2:** **Small RNA classification in human serum and plasma from uninfected and infected individuals**

**Human serum/plasma**	**Uninfected serum (European control)**	**Infected serum (Cameroon)**	**Uninfected plasma (Ghana)**	**Infected plasma (Ghana)**
**Trimmed reads**	**25519512**	**23734119**	**24992446**	**10015190**
**Human genome match**	**9998552**	**9331113**	**12936180**	**4791645**
**Unambiguous**	**9937398**	**9102817**	**12846400**	**4742963**
rRNA	15854	26512	13022	6744
tRNA	2568803	82382	6442858	1724586
Y RNA	1149988	2408124	1004222	476464
Other Rfam	198310	33516	8497	3791
miRNA	5589367	5924748	5266797	2472450
***O. volvulus*** **genome match**	**304991**	**583693**	**328590**	**157005**
**Unambiguous**	**243837**	**355397**	**238810**	**108323**
rRNA	140174	108351	132900	49797
tRNA	358	400	1884	1929
Y RNA	50	0	0	0
Other Rfam	2434	2712	3013	688
miRNA	0	75	344	743

Here we focus on the miRNAs detected in the serum from humans testing positive for *O. volvulus* that can be assigned a nematode origin. From the combined datasets we identify a total of six *O. volvulus* miRNAs. The nematode origin of these is evident from a number of criteria: 1) they map perfectly to regions that fold into hairpin structures within the *O. volvulus* genome; 2) they are not other classes of sncRNA and 3) they are not present in the sera of European controls. Of the six miRNAs identified, all were identical to the *O. ochengi* miRNAs found in nodules (Table 3). Two of the miRNAs, miR-71 and lin-4, are detected in infected samples from both Ghana and Cameroon but neither endemic nor European controls. Two are detected only in the infected pooled sample from Ghana (miR-100a, miR-87) and two of these are present in infected and endemic control samples from Ghana (miR-100d, bantam-a). As the endemic control sample was a pool of 13 individuals, it is possible that these miRNAs could derive from an individual misdiagnosed as negative for onchocerciasis or co-infected with other co-endemic nematode parasites in these regions. Of note, miR-92 and let-7 were detected in some of the libraries however the mature miRNAs are perfectly conserved between nematodes and mammals. There can be some heterogeneity in the terminal nucleotides of these miRNAs based on non-templated additions [40] (as is common for miRNAs) such that they could technically align better to parasite than host. The exact origin(s) of these miRNA cannot be inferred.

**Table 3:** **Read numbers of nematode-derived miRNAs detected in serum or plasma from individuals who tested positive for** ***O. volvulus***

**miRNA**	**RNA sequence**	**Precursor coordinates**	**Uninfected serum (European control)**	**Infected serum (Cameroon)**	**Uninfected serum (Ghana)**	**Infected serum (Ghana)**
**miR-71**	UGAAAGACAUGGGUAGUGAGAC[G]^1^	OVOC.OM1b:9907432..9907494:+	**0**	**43**	**0**	**98**
**lin-4**	UCCCUGAGACCUCUGCUGCGA	OVOC.OM4:5453650..5453708:-	**0**	**32**	**0**	**73**
**miR-100d**	AACCCGUAGUUUCGAACAUGUGU	OVOC.OM1a:1762729..1762789:-	**0**	**0**	**121**	**314**
**miR-87-3p**	GUGAGCAAAGUUUCAGGUGUUC	OVOC.OM2:17655901..17655965:-	**0**	**0**	**0**	**85**
**miR-100a**	UACCCGUAGCUCCGAAUAUGUGU	OVOC.OM1a:1763611..1763671:-	**0**	**0**	**0**	**102**
**bantam-a**	UGAGAUCAUUGUGAAAGCUAUU	OVOC.OM2:1211194..1211257:-	**0**	**0**	**223**	**71**

^1^Brackets indicate heterogeneity in the 3′ terminal nucleotide between datasets.

### Common and distinct circulating miRNA signatures in filarial infections

**Figure 3:**
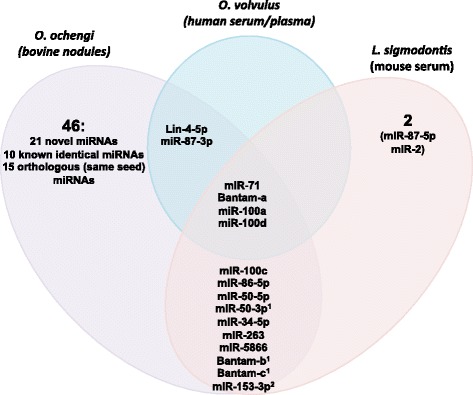
**Venn diagram depicting overlap in extracellular miRNAs identified in filarial infections.** Overlapping mature miRNAs sequences identified in cattle nodule fluids (*O. ochengi*), serum/plasma from infected patients in Cameroon and Ghana (*O. volvulus*) and infected mouse serum (*L. sigmodontis*, as previously reported [9]). ^**1**^These miRNAs differ by 1 nt outside of the seed region in *L. sigmodontis* and *O. ochengi*. ^**2**^miR-153-3p is identical to mammalian miR-153-3p from nucleotides 1–21.

**Figure 4:**
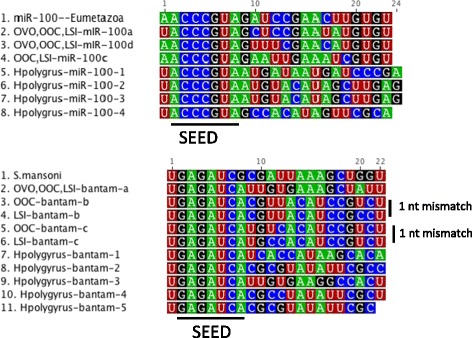
**Sequences of extracellular miR-100 and bantam family members in nematode parasites.** The conserved miR-100 sequence (Eumetazoa) is shown in relation to the nematode family members identified in these datasets: *O. volvulus* (OVO), *O. ochengi* (OOC), *L. sigmodontis* (LSI) or *H. polygyrus* (Hpolygyrus). The *S. mansoni* bantam sequence is taken from Hoy et al., PLos NTDS 2014 [4]. The naming of family members is arbitrary.

We recently identified 16 miRNAs in the serum of mice infected with the filarial nematode *L. sigmodontis* and four of these are identical to the *O. volvulus* miRNAs detected in human serum (mir-71, two miR-100 members, and one bantam family member) and one is derived from the other arm of the hairpin of a *O. volvulus* miRNA (miR-87). A further seven of the *L.sigmodontis* miRNAs are identical to *O. ochengi* miRNAs found in the nodule fluid and three (miR-50-3p and Bantam-b,c) differ by 1 nt outside of the seed region (Figure 3). Strikingly multiple miR-100 and bantam family members are present in the datasets. These also dominate the secretion product of the gastrointestinal nematode *H. polygyrus* [9]. The *O. volvulus* miR-100 and bantam miRNAs identified have distinct sequences outside of their seed regions from the miRNAs in *H. polygyrus* (Figure 4).

To compare relative quantities across the datasets and to qualify our limit of detection, we used total host miRNA read counts in the same samples as a normalizer. The individual parasite miRNAs are detected at a range of 26 to 12,863 per million bovine miRNA reads (*O. ochengi* nodule fluid), 5 to 127 per million human miRNA reads (serum/plasma from *O. volvulus* positive individuals) and 2 to 367 reads per million mouse miRNAs (serum from *L. sigmodontis* infected mice) as shown in Table 4. We anticipate these relative proportions will vary depending on the intensity of infection. Importantly, this provides a baseline for comparing relative levels of parasite miRNAs between studies and understanding the limits of detection in each study.

**Table 4:** **Relative abundance of nematode miRNA in fluids in comparison to host miRNAs (reads per million)**

**miRNA**	**Sequence**	***O. ochengi***	***O. volvulus*** **(Ghana)**	***O. volvulus*** **(Cameroon)**	***L. sigmodontis***
**Ooc-miR-71**	UGAAAGACAUGGGUAGUGAGAC[G]^3^	117.6	39.6	7.3	25.1
**Ooc-lin-4**	UCCCUGAGACCUCUGCUGCGA	1355.8	29.5	5.4	ND (<0.8)^2^
**Ooc-miR-100d** ^**1**^	AACCCGUAGUUUCGAACAUGUGU	86.2	127.0	ND (<0.3)^2^	31.4
**Ooc-miR-87-3p**	GUGAGCAAAGUUUCAGGUGUUC	26.1	34.4	ND (<0.3)^2^	ND (<0.8)^2^
**Ooc-miR-100a**	UACCCGUAGCUCCGAAUAUGUGU	979.6	41.3	ND (<0.3)^2^	376.0
**Ooc-bantam-a** ^**1**^	UGAGAUCAUUGUGAAAGCUAUU	12863.4	28.7	ND (<0.3)^2^	35.3
**Ooc-miR-86**	UAAGUGAAUGCUUUGCCACAGUCU	65.3	ND (<0.8)^2^	ND (<0.3)^2^	44.7
**Ooc-miR-263/183**	AAUGGCACUAGAUGAAUUCACGG	44.4	ND (<0.8)^2^	ND (<0.3)^2^	5.5
**Ooc-miR-50-5p**	UGAUAUGUCUGAUAUUCUUGGGUU	31.3	ND (<0.8)^2^	ND (<0.3)^2^	7.9
**Ooc-miR-34**	UGGCAGUGUGGUUAGCUGGUUGU	70.5	ND (<0.8)^2^	ND (<0.3)^2^	6.3
**Ooc-miR-5866**	UUACCAUGUUGAUCGAUCUCC[A]^3^	70.5	ND (<0.8)^2^	ND (<0.3)^2^	1.60
	**Total host miRNAs**	**382791**	**2472450**	**5924748**	**1273839**

^1^Also found in endemic Ghanaian controls.

^2^ND = not detected; the limit of detection is shown in (), based on the number of total host miRNAs sequenced and assuming 2 reads are required to identify a parasite sequence.

^3^Brackets indicate heterogeneity in the 3′ terminal nucleotide between datasets.

A recent analysis of serum from humans infected with *O. volvulus* using deep sequencing reported 20 putative *O. volvulus* miRNAs that have no overlap with those we identify here [5]. Thirteen of the reported sequences were < 17 nt long, or detected in only one read and thus are not analysed by our criteria. Of the seven additional sequences three of these perfectly align to human ribosomal RNA and two of these (PC-5p-31768_12, PC-3p-46055_7) are found as a part of longer sequences in European control serum. One of the sequences, let-7, is also found in our datasets but we do not confidently assign this to nematode origin given its conservation in the mammalian host.

## Discussion

The discovery that RNA is secreted by nematodes opens up many avenues for further investigation into their functional properties and diagnostic utility. Here we report that small noncoding RNAs derived from *Onchocerca spp*. are present in host tissues, both at a concentrated site of infection (nodule fluid) and in the circulatory system (serum/plasma) of their hosts. Six *O. volvulus* miRNAs were identified in human plasma, all of which are identical to those found in *O. ochengi* nodule fluid, and four of which are also identical to those found in serum of mice infected with the related filarial nematode *L. sigmodontis* (Figure 3). This suggests extensive overlap in the identity of extracellular parasite-derived miRNAs in filarial infections and gives confidence in the conserved nature of RNA secretion among these pathogens. This is further supported by a report published while this manuscript was in preparation which identified miRNA candidates of potential nematode origin in the plasma of baboons infected with *Loa loa* and the plasma of an *O. ochengi*-infected cow [41]: 4 of the 6 miRNAs that we identify in *O. volvulus*-infected humans are among the 22 miRNA candidates found in *Loa loa*-infected baboons and 2 of the 62 *O. ochengi* miRNAs in nodules are among the 10 candidates found in bovine plasma (Additional file 1: Table S1).

A common feature in all the infections is the presence of miR-71, bantam family and miR-100 family miRNAs (where family is defined based on identical seed sequences, nucleotides 2–8). We previously identified 5 miR-100 family members within the top 20 most abundant miRNAs secreted by *H. polygyrus* [9]. The factors dictating the expansion of this miRNA family are not known; miR-100 is one of the oldest miRNAs, having evolved in the last common ancestor of Eumetazoa (the highly conserved sequence is noted in Figure 4 and is identical across parasitic nematodes and all of their mammalian hosts). This family has expanded in some animal lineages: in *C. elegans* it is referred to as the miR-51 family and is redundantly required for embryonic development [42] and also involved in developmental timing and buccal cavity formation [42,43]. Why members of this family are secreted by parasitic nematodes is unknown and raises interesting questions regarding whether these would interact with host targets. From a diagnostic standpoint it is worth noting that the sequences outside the seed region differ between the filarial nematodes and *H. polygyrus* (Figure 4). We also identify bantam family members in serum of both *L. sigmodontis* and *O. volvulus* infected hosts. Two of the bantam family members found in the serum of *L. sigmodontis* infected mice have a 1 nt mismatch to the family members in *O. ochengi* and *O. volvulus* (Figure 4). One of the bantam members identified here appears conserved and secreted in all Clade III nematodes. We identified this miRNA in *O. ochengi* nodule fluid, serum from *O. volvulus*-infected individuals and serum from mice infected with *L. sigmodontis*. However, this miRNA sequence was also present in endemic controls from the Ghana cohort. This may represent a false negative individual or may occur if another parasite in one or more of the control individuals also secretes bantam orthologs. According to miRbase, this sequence is specific to filarial nematodes and is not present in the Clade V nematodes (Rhabditida; strongyles including *H. polygyrus*, free-living rhabditids and relatives). Interestingly, the secretion of bantam family members also occurs in trematodes; we previously identified a bantam family member (distinct in sequence from those identified here, Figure 4) in the serum of mice and humans infected with *S. mansoni* [44], and demonstrated its utility as a biomarker for schistosomiasis [4].

A key criterion in our analysis is the requirement that the annotated nematode miRNAs do not have a match in the host genome. A recent study reported putative *O. volvulus* miRNAs in human serum [5], some of which we annotate here as human ribosomal RNAs. This does not rule out that human sequences could also serve as a marker of infection, but it will be imperative to compare serum from uninfected individuals to avoid false positives and to build a better context for when and why these sequences can be detected.

We previously identified miRNAs derived from *L. sigmodontis* in mouse serum during patent infection [9]. However using the same library preparation methodology and sequencing at the same depth of coverage we did not detect miRNAs derived from the gastrointestinal nematode *H. polygyrus* in the serum of infected mice (at day 14, when the adult worms reside in the small intestine). Since the adult *H. polygyrus* worms secrete miRNAs within exosomes *in vitro* [9] it seems likely that lack of detection in serum relates to the localization of the parasite in the host. In support of this a recent report identified 245 putative parasite miRNAs in the serum of dogs infected with the heartworm *D. immitis* [5]. Individuals infected with this species of filaria would be expected to have a higher concentration of circulating parasite-derived miRNAs (relative to other filariae) due to the presence of both Mf in the blood and adult worms in the pulmonary artery and heart.

As observed in the *L. sigmondontis* dataset the *O. volvulus* miRNAs are much less abundant than host miRNAs in serum or plasma: in the Ghana samples we mapped 743 reads to 6 different nematode-derived miRNAs, compared to approx. 2.5 million human miRNAs reads in the same library. Low-abundance is a challenge when detecting any type of parasite-derived molecule in host fluid. An advantage of the miRNAs is that these can be amplified by PCR prior to detection. Nonetheless, methods for enriching parasite material are likely to be advantageous in terms of maximizing the specificity and sensitivity of detection. In this regard, we have shown nematode miRNAs are secreted within extracellular vesicles *in vitro* [9]. Further work is required to understand whether parasite and host miRNAs exist in similar or distinct complexes in host fluids, and/or whether these can be further purified prior to RNA extraction to reduce the scope for cross-contamination.

The biology and dynamics of secreted miRNAs are thus very open topics. Nothing is known at present about the extent to which different parasite life stages secrete miRNAs or what their half-lives are in host tissues including blood. We anticipate that the diagnostic utility of these molecules will also depend on the degree to which RNA secretion is regulated, the mechanism by which the small RNAs enter circulation and the stability of each RNA species in different fluids.

## Conclusions

We have identified a total of 62 miRNAs derived from *O. ochengi* in bovine nodule fluids, including miRNAs that are perfectly conserved in other filarial nematodes and some that do not have homology to other nematodes. Six of the conserved miRNAs are present in serum or plasma from humans testing positive for *O. volvulus* in Cameroon and Ghana. Four of these are also identical to those found in the serum of mice infected with *L. sigmodontis* including miR-100 and bantam family members. These findings support the conserved nature of RNA secretion by nematode parasites and identify miRNAs as a new potential biomarker for filarial infections that could significantly improve the diagnostic outlook for these neglected conditions. Further studies investigating exactly which parasite life stage(s) secrets such miRNAs, their stability, half-lives and localization within the host will drive forward the applications of parasite-specific miRNAs as biomarkers for onchocerciasis.

## Additional files

Additional file 1: Table S1. miRNA candidates found in *O.ochengi* nodules and comparison to *Loa loa* and *O.ochengi* miRNA candidates reported in Tritten et al., Molecular & Biochemical Parasitology 2014 [41]. Additional file 2: Figure S1. Small RNA profile of human serum comparing two different extraction kits. Gel of total RNA (1 uL) extracted from three replicates of human European control serum using two different kits: miRNeasy serum/plasma kit (Qiagen) and miRCURY RNA Isolation kit Biofluids (Exiqon) based on small RNA chip (Bioanalyzer).
