# Supplementary figures and images for: Extracellular Onchocerca-derived small RNAs in host nodules and blood
Source: Parasit Vectors. 2015 Jan 27;8:58. doi: 10.1186/s13071-015-0656-1 (PMC4316651; doi:10.1186/s13071-015-0656-1)

Supplementary figure 1

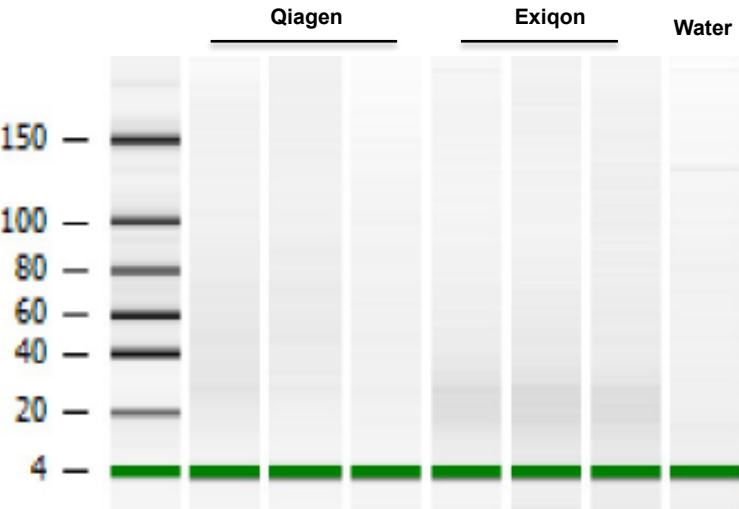

Supplement: Additional file 1: Table S1. — miRNA candidates found in O.ochengi nodules and comparison to Loa loa and O.ochengi miRNA candidates reported in Tritten et al., Molecular & Biochemical Parasitology 2014 [41]. [file 13071_2015_656_MOESM1_ESM.pdf]
